# Cardiometabolic Plasticity in Response to a Short-Term Diet and Exercise Intervention in Young Hispanic and NonHispanic White Adults

**DOI:** 10.1371/journal.pone.0016987

**Published:** 2011-02-22

**Authors:** Stacy L. Schmidt, Matthew S. Hickey, Kathryn M. Koblenz, Holly Klamer, Maria F. Botero, Kyle T. Pfaffenbach, Michael J. Pagliassotti, Christopher L. Melby

**Affiliations:** 1 Nutrition and Metabolic Fitness Laboratory, Department of Food Science and Human Nutrition, Colorado State University, Fort Collins, Colorado, United States of America; 2 Human Performance/Clinical Research Laboratory, Department of Health and Exercise Science, Colorado State University, Fort Collins, Colorado, United States of America; 3 Molecular Nutrition Laboratory, Department of Food Science and Human Nutrition, Colorado State University, Fort Collins, Colorado, United States of America; University of Tor Vergata, Italy

## Abstract

**Background:**

Young adult Mexican Americans (MA) exhibit lower insulin sensitivity (Si) than nonHispanic whites (NHW), even when controlling for fitness and adiposity. It is unclear if MA are as responsive to the same lifestyle intervention as NHW.

**Objective:**

We developed a model to examine cardiometabolic plasticity (i.e., changes in Si and plasma lipids) in MA compared to NHW adults in response to a diet-exercise intervention.

**Design:**

Sedentary subjects (20 NHW: 11F, 9M, 23.0 y, 25.5 kg/m^2^; 17 MA: 13F, 4M, 22.7 y, 25.4 kg/m^2^) consumed their habitual diets and remained sedentary for 7 days, after which fasting blood samples were obtained, and a 3-h intravenous glucose tolerance test (IVGTT) was performed with the insulin area under the curve (IAUC) used to estimate Si. Subjects then completed a 7-day diet/exercise intervention (diet: low saturated fat, low added sugar, high fiber; exercise: cycling, six total sessions lasting 40–45 min/session at 65% VO_2_ max). Pre-intervention tests were repeated.

**Results:**

Pre intervention IAUC was 28% higher (p<0.05) in MA (IAUC pre  =  2298 µU*180 min/mL) than in NHW (IAUC = 1795 µU*180 min/mL). Following the intervention, there was a significant reduction in IAUC in MA (29%) and NHW (32%), however, the IAUC remained higher (p<0.05) for MA (post  = 1635 µU*180 min/mL) than for NHW (post = 1211 µU*180 min/mL). Pre test plasma lipids were not different in MA compared to NHW. Plasma cholesterol and TG concentrations significantly improved in both groups, but concentrations of low density lipoprotein-cholesterol and small dense LDL particles significantly improved only in the NHW.

**Conclusion:**

With a short-term diet-exercise intervention, the magnitude of improvements in Si and serum cholesterol and TG in Hispanics are similar to those in NHW. However, because at the outset MA were less insulin sensitive compared to NHW, within the short timeframe studied the ethnic gap in insulin sensitivity remained.

## Introduction

Hispanic Americans, the largest and fastest growing minority group in the U.S., have a disproportionately high prevalence of overweight and obesity, Type 2 Diabetes (T2D) and the metabolic syndrome (MetS) compared to non-Hispanic whites (NHW) [1], [2]. Hispanics also exhibit substantially higher levels of insulin resistance compared to NHW, even among nonobese, young adults [3], [4], [5], foreshadowing their greater future burden of T2D and its complications. Insulin resistance is strongly correlated with high plasma triglycerides, small dense LDL particles, as well as low HDL levels [6], [7], [8], [9], [10], [11], [12].

The source of this ethnic disparity is unclear, but both genetic and environmental factors are likely contributors [13]. It is hypothesized that lifestyle factors (e.g. lower levels of physical activity and an energy-rich diet) leading to excess adiposity are the primary environmental factors related to the greater occurrence of T2D and the MetS among Hispanics. Several studies have shown total dietary fat, saturated fat and sugar intake in Mexican Americans (MA), the largest subgroup of U.S. Hispanics, to be high, with relatively low intakes of fruits, vegetables, and dietary fiber [14], [15]. As MA become more acculturated to the U.S., their leisure time physical activity decreases and there is an increased intake of saturated fats and refined carbohydrates [16], [17]. Not surprisingly, as MA transition from their cultural norms to a more Americanized lifestyle, their risk for developing T2D and the MetS increases substantially, suggesting that MA may have a lower tolerance for the unhealthy environmental factors present in the United States [18], [19].

Despite the notion that less healthy diets and low levels of physical activity are the major contributors to T2D and the MetS in MA, we have previously shown that even after matching for cardiorespiratory fitness, body composition, and body fat distribution, young MA adults still exhibit lower insulin sensitivity and greater markers of sub-clinical inflammation than their NHW counterparts, [3], [20]. This finding suggests that genetic and/or epigenetic factors may play a large role in risk for T2D and the MetS and presents the possibility that similar lifestyle habits may produce differential phenotypic responses across ethnic and racial groups. For example, Monda et al [21] recently showed that regular exercise was significantly related to lower TG in NHW, but not in African Americans, suggesting that response to health-related behaviors may differ by race or ethnicity. Experimental evidence from the Diabetes Primary Prevention Trial (DPPT) suggests that lifestyle changes (diet, regular exercise, and weight loss) can favorably affect risk for diabetes among pre-diabetic Hispanic adults despite the apparent genetic predisposition [22]. However, the contribution of changing the habitual dietary intake pattern and physical activity on diabetes risk cannot be determined independent of the substantial weight loss that also occurred among study participants [23].

Short-term increases in physical activity, independent of any substantial training adaptations, weight loss, and changes in body composition, have produced beneficial modifications in skeletal muscle protein expression and improved insulin sensitivity and circulating TG, all of which improve risk for T2D and MetS [24], [25], [26], [27]. Seven days of exercise have been shown to improve insulin sensitivity in NHW subjects [24], [28], [29].

Given the high prevalence of T2D and MetS in MA, we developed a 7-day diet-exercise intervention to compare its effects on cardiometabolic plasticity in young, previously sedentary, non-obese MA and NHW adults. The term cardiometabolic plasticity indicates the degree of response in metabolic parameters, such as insulin sensitivity and blood lipids, to the lifestyle intervention. We hypothesized that the MA compared to NHW would exhibit lower initial insulin sensitivity and greater dyslipidemia, but would experience intervention-induced improvements in these parameters. We were interested in determining whether or not the gap in insulin sensitivity between MA and NHW would be narrowed, remain the same, or even be widened in response to both groups participating in the identical diet-exercise intervention.

## Materials and Methods

### Ethics Statement

Consent was obtained from each volunteer prior to testing, and the study protocol and ethical standards employed were approved by the Colorado State University Human Research Committee.

### Subjects

A total of 37 Mexican-American (4 male, 13 female) and non-Hispanic white (9 male, 11 female) subjects aged 18–39 participated in this study (Table 1). Subjects were sedentary, non-smoking, with no known diseases. Subject inclusion criteria were: BMI <30 kg/m^2^, fasting blood glucose <110 mg/dl, blood pressure <140/90 mmHg, VO_2_max of <35 ml/kg/min for females and <45 ml/kg/min for males, and no history of endocrine disorders. Individuals were excluded if they were pregnant, used tobacco, had diabetes, reported a history of eating disorders, were vegetarians, exercised more than once a week for the past 6 months, or used medication that could influence insulin signaling or plasma lipids. Females using hormonal contraceptives were allowed to participate in the study in order to ease recruitment difficulties, despite the possibility that the progestional agent might influence baseline insulin sensitivity [30]. However, both pre- and post-test measures occurred during the active phase of hormonal contraceptive use to minimize any confounding. Women not using oral contraceptives completed pre- and post-testing in the mid-follicular phase of their menstrual cycles. Subjects were weight stable (±2.5 kg) for the previous six months. Mexican-American subjects verified that at least 3 of their grandparents were of Mexican descent, and NHW subjects traced their ethnicity to at least 3 grandparents having Anglo-European ancestry. Determination of sample size was based on our previous study in which we detected a difference in insulin sensitivity between 13 MA and 13 NHW study participants, and the study by Houmard et al in which insulin sensitivity was improved by 7 days of cycling exercise in nine sedentary men [3], [24].

**Table 1:** Baseline Characteristics of Study Participants.

		NHW(n = M, 11F)	MA(n = 4M, 13F)
Age (years)	Males	21.8 (2.4)	25.5 (5.9)
	Females	24.0 (5.2)	21.8 (5.3)
	Combined ^a^	23.3 (1.3)	23.4 (1.5)
Weight (kg)	Males	86.0 (10.1)	84.1 (2.4)
	Females	71.2 (9.6)*	64.7 (5.4)
	Combined ^a^	76.2 (1.9)#	73.0 (2.1)
Height (cm)	Males	181.2 (8.3)	178.0 (4.3)
	Females	168.9 (6.4)*	160.6 (4.8)
	Combined ^a^	173.8 (1.7)#	166.3 (1.9)
BMI (kg/m^2^)	Males	26.2 (1.7)	26.5 (1.2)
	Females	25.0 (2.5)	25.0 (2.0)
	Combined ^a^	25.4 (0.5)	25.7 (0.6)
% body fat	Males	20.4 (2.6)	23.1 (0.5)
	Females	35.0 (5.3)	36.3 (5.4)
	Combined ^a^	29.3 (1.1)	31.1 (1.2)
Waist (cm)	Males	86.5 (5.4)	89.5 (1.3)
	Females	76.8 (8.6)	79.1 (6.3)
	Combined ^a^	80.1 (1.7)	83.6 (1.8)
VO_2_ Max (ml/kg/min)	Males	38.9 (5.3)	37.7 (4.1)
	Females	29.4 (4.1)	31.2 (3.5)
	Combined a	33.8 (1.0)	34.2 (1.1)

Values are means (SD).

a  =  Adjusted means using sex as a covariate (SE).

* =  significantly different from same sex MA (p<0.05).

#  =  significantly different from MA (p≤0.05).

### Study Design

After completing initial screening tests, eligible participants spent a baseline period of 7 days consuming their normal diets and refraining from any formal exercise, as was their custom. Subjects recorded their food intake for 3 consecutive days. Immediately following the baseline period, subjects underwent an intravenous glucose tolerance test (IVGTT). For the next 7 days, study participants exercised for 6 of the 7 days and ate only the food that was prepared for them by the study investigators. Following the 7-day intervention, subjects underwent a second IVGTT.

### Specific Procedures

#### Anthropometric measures

Body weight was measured on a physicians balance beam scale (Health o meter, Alsip, IL) to the nearest 0.1 kg. Body height was measured with a wall-mounted stadiometer to the nearest 1.0 mm. Waist and hip circumferences were measured using a non-stretching tape measure to the nearest 0.1 cm with subjects in a standing position. The percentage body fat, absolute fat mass, and fat-free mass were measured using dual-energy x-ray absorptiometry (DEXA) (Model DPX-IQ Lunar Corp., Madison, WI). Medium length scans (20 min) were used for all subjects.

#### Resting Energy Expenditure (REE)

REE was measured for the purpose of estimating each subject's daily energy requirement, which was then used to provide subjects with the appropriate estimated energy intake during the 7 day protocol to keep them in energy balance. Subjects arrived at the lab after a 12-h overnight fast and before engaging in any type of physical activity. Indirect calorimetry (CPX Express, Sensor Medics, St. Paul, MN) was used to establish REE. Subjects rested in a supine position for 20 minutes with a mouthpiece and nose clip, while resting VO_2_ and VCO_2_ values were obtained by open circuit spirometry. The final 10 minutes of data were used for determination of REE using the Weir equation [31].

#### Cardiorespiratory Fitness

Maximal oxygen consumption (VO_2_max) was measured to determine cardiorespiratory fitness. VO_2_max was measured via incremental cycling exercise to volitional exhaustion in each subject. Oxygen consumption, carbon dioxide production, pulmonary ventilation, and the respiratory exchange ratio (RER) were determined with computer assisted open circuit spirometry (ParvoMedics TrueOne® 2400, Salt Lake City). Subjects cycled at a constant revolutions per minute (70–90), while the resistance was gradually increased until volitional exhaustion. Workload commenced at 50 W for two minutes and was increased by 1W every 3 seconds thereafter. To ensure maximum effort was achieved, two of the following criteria had to be fulfilled: whole body respiratory quotient >1.1, achieved maximum heart rate within 5% of the age-predicted maximum, and/or an increase in oxygen consumption in response to the final workload of <2.0 ml/kg body wt/min.

#### Dietary Assessment

To determine the average habitual energy and macronutrient intake, a detailed 3-day diet record was obtained from all subjects over 2 weekdays and 1 weekend day during the one-week baseline period. Subjects were instructed on the procedures for recording food intake accurately based on determination of specific serving sizes. The Food Intake Analysis Software (FIAS version 3, University of Texas Health Sciences Center, School of Public Health, 1998) was used to analyze total energy and macronutrient intake of each subject's 3-day diet.

#### Insulin Sensitivity

Insulin sensitivity was estimated using an intravenous glucose tolerance test (IVGTT) for insulin and glucose area under the curve over the 3-h post glucose infusion. Subjects reported to the health center on the campus of Colorado State University following an overnight fast. An intravenous catheter was placed in an antecubital vein for the collection of blood samples and administration of glucose. Two fasting blood samples were taken 5 minutes apart to determine plasma glucose and insulin concentrations, and insulin sensitivity was estimated from these fasting samples via the homeostasis model assessment of insulin resistance (HOMA-IR). The second sample was followed by a 90-second glucose infusion, in the form of a 50% dextrose solution using a relative dose based on body size (0.3 g/kg). The catheter was then flushed with approximately 10 ml of normal saline solution. Blood samples were collected in tubes containing EDTA at 2, 4, 8, 19, 22, 30, 40, 50, 58, 63, 70, 100, 140, and 180 min following glucose infusion. Blood tubes were centrifuged at 4°C and plasma was stored at −70°C until assayed. The area under the 3-h response curve (AUC) was calculated for glucose and insulin based on the trapezoidal method [32]. HOMA-IR was determined: HOMA-IR  =  [insulin (µU/mL) x glucose (mmol/L)/22.5]. Our intention was to utilize the minimal model for analysis of glucose and insulin curves to calculate values for insulin sensitivity; however, we were not able to utilize this index due to the absence of insulin augmentation and the inadequacies of the model in calculating insulin sensitivity with the reduced sampling schedule. We chose not to augment with exogenous insulin due to the risk of inducing hypoglycemia in these healthy, non-diabetic subjects.

#### Dietary Intervention

During the experimental phase, subjects were provided with all meals, snacks, and beverages for 7 consecutive days. Meals were prepared and weighed by study investigators to the nearest gram. Meals consisted of a variety of fruits and vegetables, whole grains, non-fat and low fat dairy products, legumes, and lean meats. The diets were designed to be low in both saturated fat (less than 5% energy intake) and refined carbohydrates (refined grains and added sugars) and rich in dietary fiber. Daily energy intake for each study participant for the seven day dietary intervention was based on the following: (REE x 1.3) + (net cost of exercise) + 200 kcal food modules as desired [33]. The food modules had the same macronutrient content as the entire experimental diet and were available to ensure adequate subject satiety. Prepared foods were sent home with the subjects every 2 days with instructions for consumption. Subjects were asked to consume all their food, but if unable, to return any uneaten portions if they could not consume all the food. None of the subjects reported having inadequate food available and only one subject consumed the additional food modules.

#### Exercise Intervention

Subjects exercised 6 sessions in 7 days following the baseline IVGTT. The 6 sessions consisted of stationary cycling for 40 minutes at a heart rate that elicited 65% of VO_2_max for the first three exercise bouts, and 45 minutes for the last three bouts over the 7 day period. Subjects completed no more than one exercise session in a day and always completed their final exercise session the day prior to the post-intervention measurements, which were completed 7 days following the baseline measurements. To determine the heart rate corresponding to each subject's work intensity at 65% of VO_2_ max, oxygen consumption and heart rate were measured simultaneously with subjects cycling on a stationary cycle ergometer during the first exercise session. Intensity was gradually increased until oxygen consumption was stabilized at 65% of VO_2_max. Steady state heart rate at 65% of VO2 max was determined, and this became the target heart rate for each of the subsequent exercise bouts for the remainder of the intervention. For all exercise sessions, subjects were supervised by study investigators to ensure that the proper heart rate was maintained for the duration of each bout. The final IVGTT was performed ∼17 hours after the final exercise session and 12 h following consumption of the last meal provided.

#### Plasma Assays

Plasma samples were stored at −70°C until analysis. Glucose concentrations were measured using the glucose oxidase method on an automated glucose analyzer (YSI 2300, YSI Inc. Yellow Springs, OH). Plasma C-peptide concentrations were measured by the University of Colorado Denver Clinical and Translational Research Center (CTRC) using a Siemens Competitive Radioimmunoassay (Siemens Healthcare Diagnostics, Inc. Los Angeles, CA). Plasma insulin concentrations were measured by the CTRC using a simultaneous one-step immunoenzymatic (“sandwich”) assay for use with the Beckman-Coulter Access Immunoassay System (Beckman Coulter, Inc. Fullerton, CA). High density lipoprotein-cholesterol (HDL-C), (plasma triacylglycerol (TG), and low-density lipoprotein (LDL) size were measured by NMR spectroscopy (LipoScience, Inc. Raleigh, NC). LDL-C and total cholesterol were analyzed by an enzymatic colorimetric kit (Wako Chemicals USA, Inc. Richmond, VA).

#### Statistics

SPSS version 16.0 (Cary, NC) was used for data analysis. A repeated measures analysis of covariance (ANCOVA) was used to examine pre and post-intervention differences in insulin sensitivity and lipid measures, with P-values identified for interactions and main effects of ethnicity (MA, NHW) and time (pre, post intervention). Because of the differences in the proportion of males and females within each ethnic group, sex was used as a covariate in these analyses. Pairwise comparisons were examined on the adjusted means to determine if the diet-exercise intervention significantly affected insulin sensitivity and lipid measures. Values for both sexes are reported as means and standard deviations, with the combined values within ethnicity reported as adjusted means and standard errors. Statistical significance was set at p<0.05 using a one-tailed test based on the directional research hypothesis of MA being more insulin resistant than NHW, established from our previous research study [3].

## Results

### Subject Characteristics

As shown in **Table 1**, subjects were young males and females, with NHW and MA subjects similar in age, fitness level, BMI, and percent body fat. MA were significantly shorter in stature and weighed less than NHW. **Table 2** shows that total energy and macronutrient intakes based on three-day self-reported diet records were not significantly different between the two ethnicities, although female MA consumed a higher percentage of calories from fat compared to female NHW. In keeping with the goal of the experiment, the intervention diet was significantly lower in total and saturated fat and higher in total calories, protein, and fiber intake compared to subjects' reported dietary intake (**Table 2**). During the exercise sessions, subjects expended a net average of 293±88 kcals, with no differences between ethnicities (NHW: 311±97, MA: 272±73 kcals). There was a slight but statistically significant (p = 0.001) decrease in body weight from pre- to post-intervention across both groups (76.2±1.9 to 75.4±1.9 kg in NHW and 73.0±2.1 to 72.4±2.1 kg in MA).

**Table 2:** Dietary Intake.

		Baseline (Self-Reported)		Intervention	
		NHW(n = 9M, 11F)	MA(n = 4M,13F)	NHW(n = 9M,11F)	MA(n = 4M,11F)
Total kcal	Males	2418 (325) †	2655 (708)	2940 (274)	2726 (235)
	Females	2092 (712)	1653 (451)	2116 (282)	1931 (159)
	Combined ^a^	2163 (153)‡	1992(169)‡	2402 (64.7)#	2234 (71.9)
Carbohydrate (% kcal)	Males	46.6 (6.5)	46.0 (7.0)	51.0 (2.0)	51.8 (1.6)
	Females	51.4 (10.9)	46.9 (7.9) †	50.6 (1.3)	52.0 (2.4)
	Combined ^a^	48.9 (2.2)	45.3 (2.5)	51.0 (0.5)	51.6 (0.6)
Protein (% kcal)	Males	15.5 (2.2) †	13.7 (4.4) †	21.9 (0.9)	21.3 (1.7)
	Females	14.9 (3.9) †	16.1 (3.6) †	23.2 (1.2)	22.1 (1.8)
	Combined ^a^	15.2 (0.9)‡	15.9 (1.0)‡	22.8 (0.3)	22.1 (0.4)
Fat (% kcal)	Males	34.8 (5.0) †	31.7 (5.5)	27.1 (2.3)	26.9 (1.4)
	Females	29.6 (6.0)*	36.3 (6.5) †	26.2 (1.7)	26.0 (1.6)
	Combined ^a^	31.7 (1.6)‡	35.4 (1.8)‡	26.2 (0.5)	26.3 (0.5)
Sat. Fat (% kcal)	Males	12.0 (2.8) †	10.9 (2.8) †	4.9 (0.5)	4.5 (0.3)
	Females	11.2 (2.7) †	12.3 (2.4) †	4.7 (0.2)	4.8 (0.3)
	Combined ^a^	11.6 (0.6)‡	11.7 (0.7)‡	4.7 (0.8)	4.7 (0.8)
Fiber (gm)	Males	16.4 (5.0) †	15.2 (4.1) †	54.5 (6.6)	54.3 (2.8)
	Females	16.8 (6.2) †	12.6 (6.2) †	38.6 (4.8)	37.0 (3.0)
	Combined ^a^	16.0 (1.4)‡	13.1 (1.5)‡	44.5 (1.2)	42.9 (1.4)

Values are means (SD).

^a^  =  Adjusted means using sex as a covariate (SE).

* =  significantly different from same-sex MA, within study period (p<0.05).

#  =  significantly different from MA, within study period (p<0.05).

† =  significantly different from intervention, within sex and ethnicity (p<0.05).

‡ =  significantly different from intervention, within ethnicity (p<0.05).

### Pre-intervention plasma measures

**Figure 1:**
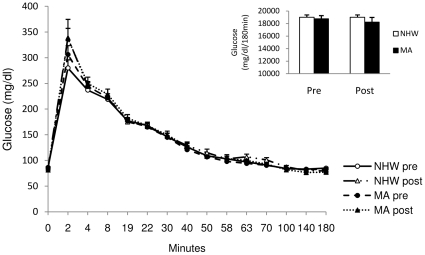
Plasma glucose area under the curve during the 3 h IVGTT for NHW and MA males and females before and after the diet-exercise intervention. Glucose AUC was not different between ethnicities pre or post intervention and did not change after the intervention. Values are means ± SE. AUC values were calculated using the trapezoidal method [32]. NHW n = 19, MA n = 14.

**Figure 2:**
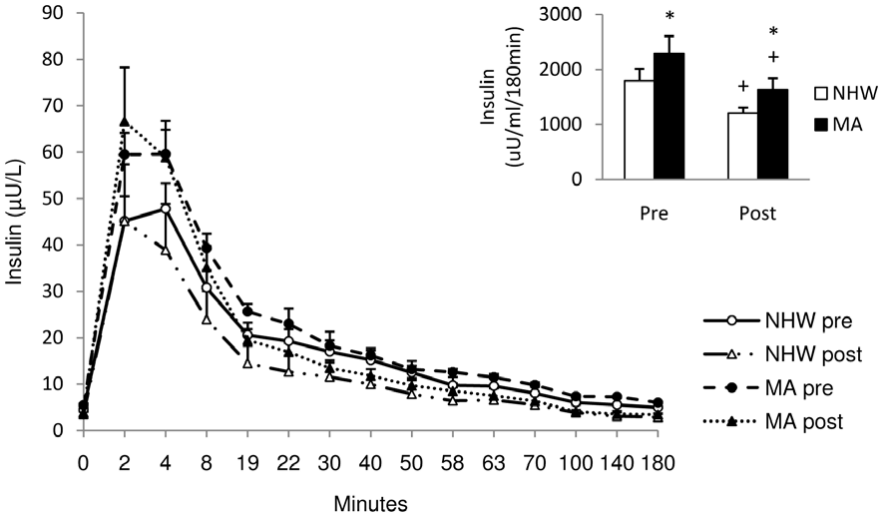
Plasma insulin area under the curve during the 3 h IVGTT for NHW and MA subjects before and after the diet-exercise intervention. Insulin AUC was significantly higher in MA subjects before and after the diet-exercise intervention and decreased significantly after the intervention in both groups. Values are means ± SE. AUC values were calculated using the trapezoidal method [32]. NHW n = 19, MA n = 14. * compared to NHW, within time period (p<0.05). + pre vs. post intervention, within ethnicity (p<0.05).

At baseline, glucose concentrations were not different between MA and NHW (**Table 3**). However, fasting insulin levels were ∼20% higher (p<0.05) in MA compared to NHW prior to the diet and exercise intervention, and HOMA-IR was also significantly higher (p<0.05) for MA, both indicating lower estimated insulin sensitivity in the MA. Fasting C-peptide values were not different at baseline between MA and NHW. The pre-intervention IVGTT revealed no significant differences in glucose area under the curve between ethnic groups (**Figure 1**). However, despite no difference in glucose AUCs for the two groups, the insulin AUC, was ∼22% higher (p<0.05) in MA compared to NHW (**Figure 2**). The measure of insulin AUC in response to glucose infusion was strongly correlated with HOMA-IR in this study (Pre IAUC with pre HOMA-IR: r = 0.85, p<0.0001, post IAUC with post HOMA-IR: r = 0.84, p<0.0001), and the pre-to-post change in the IAUC was strongly correlated with the pre-to-post change in HOMA-IR (r =  0.74, p<0.0001).

**Table 3:** Fasting Indices of Insulin Resistance.

		Pre-Intervention		Post-Intervention	
		NHW(n = 9M, 11F)	MA(n = 4M, 13F)	NHW(n = 9M, 11F)	MA(n = 4M, 13F)
Fasting Glucose	Males	90.3 (7.3)	85.3 (9.9)	91.2 (15.3)	78.3 (4.6)
(mg/dl)	Females	83.6 (6.2)	86.0 (5.5)	79.9 (4.0)	83.0 (5.6)
	Combined a	87.5 (1.8)	86.8 (2.0) ‡	85.8 (2.6)	82.1 (2.8)
Fasting Insulin	Males	5.23 (2.05)	6.88 (3.57)†	3.72 (1.66)	4.88 (2.14)
(µU/mL)	Females	3.96 (0.99)*	5.17 (2.15)†	3.25 (1.57)	4.01 (2.33)
	Combined a	4.64 (0.50)# ‡	6.34 (0.65) ‡	3.13 (0.49)	4.62 (0.54)
HOMA-IR	Males	1.20 (0.56)	1.50 (0.91)	0.83 (0.38)	0.95 (0.45)
	Females	0.82 (0.23)* †	1.12 (0.49)†	0.64 (0.29)	0.84 (0.54)
	Combined a	1.02 (0.13)# ‡	1.38 (0.14) ‡	0.71 (0.10)	0.89 (0.10)
Fasting c-peptide	Males	1.81 (0.57)†	1.93 (0.39)†	1.34 (0.51)	1.45 (0.44)
(ng/ml)**	Females	1.59 (0.34)†	1.76 (0.58)†	1.27 (0.35)	1.40 (0.54)
	Combined a	1.68 (0.12) ‡	1.82 (0.13) ‡	1.30 (0.12)	1.42 (0.13)

a  =  Adjusted means using sex as a covariate (SE).

Values are means (SD).

* =  significantly different from same-sex MA, within time period (p≤0.05).

#  =  significantly different from MA, within study period (p<0.05).

† =  significantly different from post-intervention, within sex and ethnicity (p<0.05).

‡ =  significantly different from intervention, within ethnicity (p<0.05).

**n  =  10M, 9F for NHW, n = 4M, 9F for MA pre and post-intervention.

### Post-intervention plasma measures

**Figure 3:**
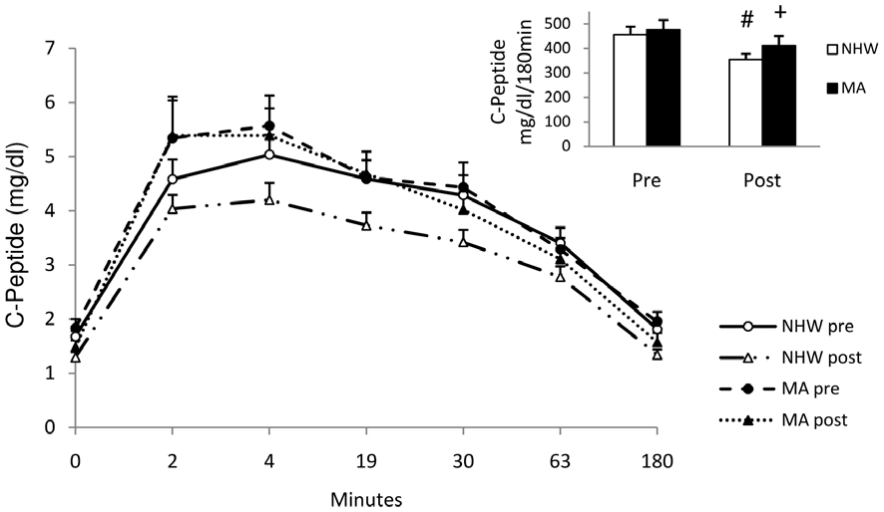
Plasma c-peptide area under the curve during the 3h IVGTT for NHW and MA subjects before and after the diet-exercise intervention. C-peptide AUC was not different between ethnicities pre or post intervention but did decrease after the intervention. C-peptide decreased after the intervention in both groups. Values are means ± SE. AUC values were calculated using the trapezoidal method [32]. NHW n = 17, MA n = 14. # pre vs. post intervention, within ethnicity (p = 0.05). ‡ pre vs. post intervention, within ethnicity (p<0.01).

The diet-exercise intervention improved estimates of insulin sensitivity in both ethnic groups. While the glucose AUC did not change following the intervention, there were significant main effects of both time and ethnicity for the IAUC. However, there was no time by ethnicity interaction indicating that while both groups improved over time, the ethnic difference remained after the intervention. Specifically, post-intervention insulin AUC remained significantly higher (∼26%) for the MA compared to NHW (p<0.05) (**Figure 2**). Fasting indices of insulin resistance followed a similar pattern, but were not significantly different in the post-intervention period (**Table 3**). MA had ∼10% higher C-peptide AUC compared to NHW following the intervention (402.6±36.4 vs. 355.8±32.8 ng/ml/180min, respectively), but this was not statistically significant (p = 0.41) (**Figure 3**). C-peptide AUC decreased in both ethnic groups, 22.0% in NHW (p = 0.05) and 14.9% in MA (p<0.01), following the intervention.

### Lipids

Pre-test lipid values did not differ between ethnicities (Table 4). There was a significant treatment effect for each of the lipids measured, indicating that all lipids decreased from pre- to post-treatment for the entire sample. There was not a significant treatment by ethnicity interaction for any of the lipids. However, all the lipid variables decreased significantly in the NHW, while for the MA, only total cholesterol, triglycerides, and HDL cholesterol significantly decreased following the intervention, with reductions in LDL-cholesterol (-7%, p = 0.07) and small, dense LDL particles (−12%, p = 0.3) not reaching significance in this group.

**Table 4:** Fasting Plasma Lipids.

		Pre-Intervention		Post-Intervention	
		NHW(n = 9M, 11F)	MA(n = 4M, 13F)	NHW(n = 9M, 11F)	MA(n = 4M, 13F)
Total Cholesterol	Males	171.3 (47.1) †	162.2 (43.4) †	149.1 (34.7)	145.0 (32.0)
(mg/dl)	Females	154.0 (45.2)	146.3 (20.4) †	145.2 (38.9)	133.2 (30.3)
	Combined a	153.6 (9.2) ‡	147.0 (10.2) ‡	139.3 (8.4)	129.3 (9.3)
LDL Cholesterol	Males	111.5 (21.7) †	108.2 (1.8)	96.4 (18.5)	97.6 (19.0)
(mg/dl)	Females	103.2 (16.3) †	100.5 (15.3)	94.3 (14.1)	97.0 (21.7)
	Combined a	105.9 (4.6) ‡	105.1 (5.1)	93.1 (4.9)	97.2 (5.5)
Triglycerides	Males	102.9 (44.2) †	118.0 (73.6)	66.6 (23.5)	59.0 (23.9)
(mg/dl)	Females	68.9 (26.0) †	81.7 (30.7) †	50.5 (28.8)	53.8 (30.8)
	Combined a	79.3 (10.1) ‡	91.7 (11.2) ‡	57.3 (6.0)	51.3 (6.6)
Small Dense LDL	Males	738.0 (267.5) †	732.0 (239.6)	518.2 (181.0)	575.5 (107.3)
particles (nmol/L)	Females	406.0 (162.3)	484.1 (315.2)	415.5 (137.1)	473.8 (254.2)
	Combined a	497.9 (48.2) ‡	594.4 (53.5)	424.2 (39.3)	523.6 (43.7)
HDL Cholesterol	Males	34.9 (7.1) †	39.8 (8.3)	31.4 (6.0)	35.3 (4.0)
(mg/dl)	Females	55.0 (13.0) †	47.5 (6.4) †	49.6 (10.9)	44.5 (6.0)
	Combined a	46.5 (2.5) ‡	43.5 (2.8) ‡	42.3 (2.1)	39.4 (2.3)

Values are means (SD).

a  =  Adjusted means using sex as a covariate (SE).

† =  significantly different from post-intervention, within sex and ethnicity (p<0.05).

‡ =  significantly different from post-intervention, within ethnicity (p≤0.01).

## Discussion

### Insulin Sensitivity

The purpose of this study was to determine whether or not MA, a high risk population for T2D and the MetS, exhibit the same degree of cardiometabolic plasticity as that of NHW (a lower risk population) in response to a short-term diet-exercise intervention. We and others (10,11,50) have shown that MA are at greater risk for insulin resistance, T2D, and the MetS relative to their age-matched NHW counterparts, even when controlling for cardiorespiratory fitness and fatness. Importantly, our data clearly show that MA exhibit metabolic plasticity such that both insulin sensitivity and plasma lipids improved significantly following the intervention. Measurement of C-peptide concentrations in plasma provides an estimate of insulin secretion. In both ethnic groups, C-peptide AUC also decreased following the intervention, which indicates that less insulin was secreted by both ethnic groups in response to the same glucose challenge, indicative of improved insulin sensitivity.

The fact that the Mexican Americans showed improvements of similar magnitude to those of the nonHispanic whites is clearly important in lowering risk for diabetes and the metabolic syndrome in this high risk group. However, a cautionary note to this positive outcome is that at least within the short timeframe studied, the improvement in insulin sensitivity in Mexican Americans did not narrow the gap in insulin resistance seen between these two groups. In fact, the 3-h insulin AUC for MA following the diet-exercise intervention was no different from the insulin AUC for NHW **prior** to the intervention. It is possible that had the intervention been extended for a longer time period, the MA would have continued to improve and possibly “caught up” to the NHW in regards to their insulin sensitivity. A longer term intervention will be required to address this issue.

Reasons for the lower insulin sensitivity in MA are not entirely clear. This phenomenon does not appear to result from any differences in the abundance of skeletal muscle insulin signaling proteins, as we have previously shown that MA and NHW do not differ in specific myocellular proteins including insulin receptor-beta, IRS-1, IRS-2, Akt, and GLUT4 [34]. Possibly the activation of these proteins via reduced phosphorylation or greater rates of dephosphorylation differs among these ethnicities, but to our knowledge this has not been examined. In a previous study [34] we found differences in insulin sensitivity were significantly attenuated after adjustment for dietary saturated fatty acid intake. Higher saturated fat intake has been linked to greater skeletal muscle accumulation of lipid metabolites including ceramides and diacylglycerols which dampen insulin signal transduction. Ethnic differences in insulin sensitivity may be related to genetic factors [35], [36], [37], but these are not well characterized. It is possible that a longer exposure to a healthy diet and regular exercise, or larger changes in exercise and dietary characteristics are required to attenuate this ethnic health disparity. While the significant diet-exercise induced improvements in insulin action among MA study participants holds promise for the prevention of diabetes in this group, research must continue to address the reasons for the ethnic differences in insulin action, as well as the exercise and dietary changes most capable of attenuating or alleviating this disparity.

### Lipids

Despite the lower estimated insulin sensitivity among MA prior to the intervention, their plasma lipid and lipoprotein profiles of the MA did not differ from the NHW. This was somewhat unexpected given that we have previously shown in NHW that lower insulin sensitivity is strongly linked to elevated plasma triacylglycerol (TG) concentrations, VLDL TGs, and concentrations of small dense LDL particles [34]. In a similar fashion, Haffner et al in the Insulin Resistance Atherosclerosis Study (IRAS) reported Hispanics to exhibit greater insulin resistance and higher TG, lower HDL-C, and smaller LDL size compared to NHW [12]. However, these subjects were older and had higher BMIs than those participants in our current study. This may suggest that lower insulin sensitivity is not linked to dyslipidemia in young MAs and/or that lower insulin sensitivity precedes the dyslipidemic phenotype, which has been previously observed in other studies [38].

The plasticity in several plasma lipid responses to the intervention in the MAs was similar to that of the NHWs. In both groups of young adults, TGs and total cholesterol were in the normal range at the outset of the study, and all decreased with only one week of an increase in physical activity and the consumption of a diet low in saturated fat and high in fiber. This short-term intervention also led to a significant decrease in HDL-cholesterol. HDL-C typically increases in response to an increase in physical activity [39], however, improvements in HDL-C are quite variable and often depend on baseline levels. Several studies have shown HDL-C to decrease with dietary changes similar to those in our intervention, and 7 days of exercise has previously been shown to induce no changes in HDL-C [28], [29], [39], [40]. The significant reduction in LDL-C and small dense LDL particles in the NHW but not in MA may reflect lower placticity in the MA compared to NHW, or may simply be a matter of a reduced power to detect such changes in the MA group owing to the smaller number of study participants.

### Strengths and Limitations

There are a number of strengths of this study: The short-term model produced significant changes in Si and plasma lipids in both groups, and thus it appears to be an effective approach to examine some aspects of cardiometabolic plasticity, independent of any substantial weight loss, and that does not require months of exercise and dietary intervention. All exercise sessions were supervised so that the intensity and duration were well-controlled across all study participants, and dietary intake was standardized for all subjects over the 7-day period.

There are a number of caveats to note: 1.) We planned to use the minimal model to assess insulin sensitivity, but decided against using insulin augmentation owing to risk of hypoglycemia in these young subjects. Unfortunately, using the minimal model algorithim, we were unable to calculate Si for a number of study participants and used the insulin area under the curve as an indicator of insulin sensitivity instead. We recognize the shortcomings of our approach. However, the dynamic measure of insulin AUC in response to glucose infusion was strongly correlated with HOMA-IR in this study, and the pre-to-post change in the IAUC was strongly correlated with the pre-to-post change in HOMA-IR. Thus, the IAUC values, along with the use of fasting insulin and HOMA-IR provide, in our judgment, sufficient data to support our contention that the MA were less insulin sensitive than NHW at the commencement of the intervention, and that insulin sensitivity improved in both groups as a result of the diet-exercise intervention. 2.) Despite our efforts to keep subjects in energy balance, there was an average weight loss of 500 g of body weight by the end of the intervention—this despite the fact that many subjects found it difficult to consume all of the food that was prepared for them. The large increase in dietary fiber owing to increased fruits, vegetables, and whole grains, as well as greater dietary protein intake are likely explanations for this phenomenon. The decrease in body weight, while quite small, may have contributed to the improvements seen in insulin sensitivity and the plasma lipids, as an acute energy deficit can enhance insulin sensitivity and lower plasma lipids [41]. However, given that both MA and NHW experienced similar changes in weight, this caveat would not appear to contribute to any differences in the ethnic responses to the intervention. We did not conduct post-intervention DEXA scans to determine the nature of the weight, i.e. loss of fat mass vs lean mass, as we did not predict a large change in body composition within one week, and the small changes seen in this study are likely within the margin of error of measurement of fat and lean tissue by DEXA. 3.) We recruited participants for our study based on body mass index (BMI) values less than 30 kg/m^2^. However based on the baseline DEXA scans, the mean percentage body fat for our participants was higher than expected and may reflect their sedentary nature. Regardless of the reason for the discrepancies between BMI and percent body fat, neither were different across ethnicities and therefore do not confound our findings. 4.) The short-term model we developed to examine cardiometabolic plasticity incorporated diet, exercise, and a small weight loss, and it is therefore impossible to determine the independent contribution of these variables. We chose this approach to maximize the possible improvements that would occur in 7 days as a means to rapidly examine potential ethnic differences in cardiometabolic response. While it is likely that with a week of lifestyle change, exercise had a greater effect on insulin sensitivity than did diet, the increase in dietary fiber may improve insulin sensitivity [42], [43], and the increase in dietary protein characteristic of our experimental diet has been shown to be a potent stimulus to improve insulin sensitivity [44]. Regardless, our standardized dietary approach across both ethnic groups enables us to rule out ethnic differences in dietary intake during the intervention as confounding factors. 5.) In addition to the beneficial effects of exercise training on insulin action, it is known that the effects on insulin sensitivity from a single bout of exercise can persist for up to 48 hours [45]. Thus changes in insulin sensitivity that we observed may very well reflect the effects of the last bout of exercise rather than the 7 day exercise intervention. While we could have used a single bout of exercise to compare ethnic responses in insulin action, we chose a 7-day period which included a standardized low saturated fat, low sugar, high fiber diet in order to examine other aspects of the cardiometabolic syndrome, including the plasma lipids and lipoproteins, which are not likely to be influenced by a single bout of exercise. 6.) There was an unequal gender distribution in this study, with fewer MA male participants than NHW males. Because males are generally less insulin sensitive than females [46], [47], the unequal gender distribution could be a confounder of our results. We dealt with this issue by covarying for gender, but recognize this approach may not entirely eliminate the confounding. Note however, that the smaller number of MA males compared to NHW males actually favors the attenuation of group differences between the MA and NHW. Had we been able to enroll more Hispanic men in the study, it is highly likely that mean difference in insulin sensitivity would have been greater between the two ethnic groups, with MA exhibiting an even lower average insulin sensitivity than NHW.

### Summary

In summary, our data show that young, sedentary MA adults exhibit lower insulin sensitivity compared to their NHW counterparts. Importantly the MA exhibit significant cardiometabolic plasticity such that both insulin sensitivity and several plasma lipids showed marked improvement in response to the short-term diet-exercise intervention. However, it is equally evident that despite similar plasticity to that of NHW in regard to insulin sensitivity, within the very short time frame studied, the MAs fell short of ‘catching up’, and the ethnic disparity in insulin sensitivity remained in the face of the same short-term lifestyle intervention. It remains unclear if a longer term intervention could completely eliminate this ethnic disparity. Research must continue to address the most effective means of eliminating this disparity in order to lessen the excess burden of T2D and the MetS in this high risk population. Potential areas of focus to eliminate this disparity include identifying and modifying the most detrimental as well as beneficial dietary patterns in the MA population, and determining the most beneficial exercise and diet prescription for MA to improve both insulin sensitivity and plasma lipids.
